# The applicability of pig oral fluid in laboratory diagnostics of porcine reproductive and respiratory syndrome and its effectiveness in controlled exposure of gilts

**DOI:** 10.3389/fvets.2026.1795728

**Published:** 2026-03-06

**Authors:** Jan Plut, Marek Brabec, Marina Štukelj

**Affiliations:** 1 Clinic for Ruminants and Pigs, Clinic for Reproduction and Large Animals, Veterinary Faculty, University of Ljubljana, Ljubljana, Slovenia; 2 Department of Statistical Modelling, Institute of Computer Science, The Czech Academy of Sciences, Prague, Czechia

**Keywords:** disease control, gilt acclimatization, GLMM, molecular diagnostic methods, oral fluid, PRRS virus, random effect models, serological methods

## Abstract

**Background:**

Porcine reproductive and respiratory syndrome (PRRS) remain a major challenge for swine health and production, and effective control depends on successful acclimation of replacement gilts. Natural exposure is commonly used but offers limited control over infection timing and immune response. This study evaluated the use of PRRS virus (PRRSV)–contaminated oral fluid (OF) ropes as an additional or sole acclimation tool and assessed the usefulness of serum and OF for monitoring PRRSV infection.

**Methods:**

Ninety-six (96) PRRSV-naïve replacement gilts were allocated to six groups (16 gilts each) and exposed to PRRSV by natural contact with infected pigs, natural exposure combined with OF-contaminated ropes, or OF-contaminated ropes alone. PRRSV RNA and anti-PRRSV antibodies were monitored weekly for 9 weeks using RT-PCR and ELISA in individual serum samples and group OF samples.

**Results:**

PRRSV infection and seroconversion occurred in all gilts exposed through natural contact, whereas exposure to OF-contaminated ropes alone did not result in consistent infection or acclimation. Gilts receiving additional exposure via contaminated ropes showed significantly earlier detection of viremia and more gilts were deemed non-viremic sooner compared with gilts exposed only by natural contact. Significant differences in antibody responses between treatment groups were observed at several time points. Detection of PRRSV RNA and antibodies in OF generally reflected serum results, although variability in antibody detection in OF was noted.

**Conclusion:**

Supplemental exposure using PRRSV-contaminated OF accelerates infection and immune response during gilt acclimation when combined with natural exposure but is insufficient as a standalone method. Oral fluid sampling represents a practical tool for monitoring PRRSV acclimation under field conditions.

## 1 Introduction

Porcine reproductive and respiratory syndrome (PRRS) is one of the most economically important diseases affecting pig production worldwide. The disease and its causative agent, porcine reproductive and respiratory syndrome virus (PRRSV), were first described in the late 1980s (1, 2). PRRS is currently present in all major pig-producing countries (3–5). Economic losses attributed to PRRS are estimated at approximately USD 664 million annually in the United States (6) and EUR 1.5 billion per year in the European Union (7). These losses are primarily associated with increased herd mortality, particularly in nursery pigs, reduced feed efficiency, reproductive failure, and secondary disease outbreaks resulting from PRRSV-induced immunosuppression (8).

PRRSV belongs to the genus *Betaarterivirus* within the family *Arteriviridae* (9). The virus is enveloped and contains a positive-sense single-stranded RNA genome (10). PRRS spreads rapidly within and between herds, predominantly through the movement of live animals and semen (11). PRRSV can survive up to 155 h at 4 °C (12) and for several months frozen at −20 °C (13).

From a pathogenesis standpoint, early systemic viremia is followed by the development of humoral responses, with virus and antibodies detectable not only in serum but also in oral and nasal secretions, which provides a biological basis for using oral matrices in surveillance and management. Once PRRS is confirmed in a herd, disease control strategies are commonly implemented to reduce production losses. Under controlled conditions, herds coexist with PRRSV, while management and biosecurity measures aim to improve overall herd health and maintain economically viable production. Effective control is associated with reduced horizontal and vertical transmission, decreased morbidity and mortality, and the development of herd immunity against the farm-specific PRRSV strain (14).

Replacement gilts represent a critical category for successful PRRS control (15). Introduction of naïve, non-immune gilts directly into an infected breeding herd frequently results in acute outbreaks and further virus transmission. Consequently, gilts must undergo an acclimation process prior to herd entry. During acclimation, replacement gilts are typically housed in gilt development units (GDU), where they become infected with PRRSV, recover, eliminate the virus, and develop homologous immunity to the farm-specific strain (16). In addition to acclimation techniques such as serum inoculation (17) and vaccination (18), a commonly used method involves exposure to infected animals—most often viraemic weaning pigs—and to infectious materials such as blood, feces, urine, oral fluid (OF), and other excreta (19). Pig OF has proven to be an efficient and practical sample for PRRSV detection and is easily collected from groups of pigs using undyed 100% cotton ropes (20). OF represents a useful alternative sample type for monitoring the acclimation process in replacement gilts. Building on its established role as a diagnostic matrix for both molecular (RT-PCR) and serological (ELISA) detection, the presence of PRRSV RNA and PRRSV-specific antibodies in oral secretions supports the concept that OF can inform both infection status and immune response during acclimation.

In the present study, we aimed to extend the use of OF-soaked ropes beyond diagnostics and evaluate their potential as a more standardized exposure method for infecting and acclimating naïve gilts in the GDU. A major limitation of this approach is the lack of precise information regarding the timing of exposure, the onset of infection, and the development of immunity in gilts. Accordingly, our hypothesis was that (i) supplementing natural exposure with OF-soaked ropes would increase the likelihood and earliness of PRRSV contact, leading to earlier detection of viremia and earlier seroconversion, and (ii) OF-based sampling (molecular and serological) would provide a practical framework for monitoring infection and immune dynamics during acclimation, given the known detection of virus and antibodies in oral secretions (20). We further posited that OF-soaked ropes used alone might be insufficient to consistently establish infection under field conditions, reflecting variability in viral load and stability in oral matrices.

The objective of this study was to evaluate gilt acclimation following exposure to different PRRSV sources and to assess whether OF exposure could accelerate virus contact, immune response, and acclimation compared with natural exposure alone. This objective directly tests the stated hypothesis by comparing natural exposure, natural exposure plus OF-soaked ropes, and OF-soaked ropes alone, while leveraging OF and serum as complementary matrices to track PRRSV infection and antibody development.

## 2 Materials and methods

### 2.1 Replacement gilts

A total of 96 replacement gilts were included in this study and divided into six groups of 16 gilts each, identified by Roman numerals I–VI. The first part of the study was conducted on a large PRRS-positive commercial farm housing approximately 3,000 breeding sows. Each week, 16 naïve replacement gilts, approximately 12 weeks of age and weighing around 30 kg, were introduced into the GDU and subjected to the routine 3-month acclimation procedure. Replacement gilts were imported from a farm with documented PRRS negative status and were sampled for blood and OF to confirm absence of PRRSV and specific antibodies upon arrival. During this period, gilts were exposed to older gilts already present in the GDU, as well as to viraemic weaning pigs and their excreta, primarily manure. Four consecutive groups of gilts (groups I–IV) were observed in this GDU for a period of 9 weeks each. All gilts were individually marked with a numbered ear tag immediately upon arrival. The gilts were housed in slate-fenced pens that allowed contact with older groups, and 10–16 viraemic weaners were mixed into the pens.

The final two groups (groups V and VI) consisted of naïve replacement gilts housed in clean, disinfected facilities at the Veterinary Faculty of the University of Ljubljana. Within the faculty building, three pens (6.5 m^2^ each) were available, housing five or six gilts per pen originating from a PRRS-negative farm.

### 2.2 Method of gilt acclimatization

The first two groups of gilts (groups I and II) were exposed to PRRSV exclusively through natural contact with infected pigs, their excreta, and aerosol transmission within the GDU. For the subsequent two groups (groups III and IV), the same acclimatization protocol was followed, with the additional use of cotton ropes impregnated with OF, collected from PRRSV-infected animals from previous groups in the same GDU. In groups III and IV, clean dry ropes were set up first, with the purpose of gaining OF sample and after the collection of the sufficient amount of OF, These ropes were removed and replaced with ropes collected and stored from the previous gilt groups and were left suspended from a crossbar in the center of the pen for 1 week, until the next sampling cycle.

Ropes were provided from the day of arrival for a total of 4 weeks and replaced weekly. The gilts readily interacted with the ropes by sniffing and chewing on them, and competition for access to the ropes was occasionally observed.

The final two groups (groups V and VI), housed in PRRSV-free facilities, were exposed solely to OF-soaked ropes obtained from PRRS-positive animals from the commercial farm GDU, in an attempt to infect and acclimate the gilts using the contaminated cotton ropes alone. For group V, ropes were stored frozen at −20 °C in clean plastic zip-lock bags prior to use. For group VI, ropes were transported directly from the GDU in a cooled container at 4 °C, with a transport time of approximately 60 min by car. In both groups, ropes were provided for 4 weeks from the day of arrival and replaced weekly.

### 2.3 Sampling and biological material

Individual blood samples and pen based OF samples were collected weekly, starting on the day of arrival of each new group of gilts (day 0). For OF samples, five ropes from a commercial oral fluid collection kit (IDEXX, Westbrook, ME, USA) were placed in each pen, and after 30 min of exposure, the collected OF was manually squeezed into 50-mL plastic containers with screw caps.

After the sampling, ropes were left in the pens for another 30 min for the gilts to chew on, so they resoaked with OF, following by the storage in clean zip-lock plastic bags and subsequently used for acclimation of groups III–VI. Individual blood samples were collected from the cranial vena cava using sterile 4–8 cm needles and transferred into sterile, individually labeled 10-mL serum tubes containing silica clot activator. Samples were stored in refrigerated containers at 4 °C and transported to the laboratory.

Oral fluid samples were centrifuged immediately at 2,000 × g for 10 min, after which the supernatant was transferred into sterile 1.3-mL vials and stored at −20 °C for serological testing and −80 °C for molecular analyses. Blood samples were centrifuged at 3,000 × g after clot formation, and serum was aliquoted and stored under the same conditions as OF samples.

Ropes containing OF intended for further use in acclimation were stored at −20 °C for groups III–V or used fresh without prior freezing for group VI. Any in between transportation of the ropes was performed in cooled environment (2–8 °C). In total, 728 individual serum samples and 50 group OF samples were collected (Table 1).

**Table 1:** The number of samples obtained.

		Number of individual serum samples	Number of group of samples
Gilt group number	I	136	9
	II	120	9
	III	128	9
	IV	120	9
	V	112	7
	VI	112	7
	Total	728	50

### 2.4 Molecular and serological testing

The total number of molecular and serological tests performed on the collected samples is shown in Table 2.

**Table 2:** The number of samples tested by different diagnostic methods.

		Number of tests with different methods			
		RT-PCR in serum	Ab ELISA in serum	RT-PCR in OF	Ab ELISA in OF
Gilt group number	I	136	136	9	9
	II	120	120	9	9
	III	128	128	9	9
	IV	120	120	9	9
	V	0	112	7	7
	VI	0	112	7	7
	Total	504	728	50	50

#### 2.4.1 Nucleic acid extraction

Viral RNA was extracted manually using the QIAamp Viral RNA Mini Kit (Qiagen, Hilden, Germany) according to the manufacturer’s instructions. RNA was isolated from 140 μL of sample supernatant and eluted in a final volume of 60 μL.

#### 2.4.2 RT-PCR for PRRSV detection

PRRSV RNA was detected using one-step RT-PCR with the One-Step RT-PCR Kit (Qiagen, Hilden, Germany) and PRRSV-specific primers (21). Each 25 μL reaction contained 5 μL of 5 × PCR buffer, 11 μL of DNase/RNase-free water, 1 μL of 10 mM dNTP mix, 0.5 μL of each primer (20 pmol/μL), 1 μL of RT-PCR enzyme mix, and 6 μL of RNA template.

Amplification was performed using a Mastercycler Nexus Gradient thermocycler (Eppendorf, Hamburg, Germany) with the following conditions: reverse transcription at 50 °C for 30 min, initial denaturation at 94 °C for 15 min, followed by 40 cycles of denaturation at 94 °C for 30 s, annealing at 60 °C for 30 s, and elongation at 72 °C for 1 min, with a final elongation step at 72 °C for 10 min. PCR products were visualized by electrophoresis on a 1.8% (w/v) agarose gel.

#### 2.4.3 ELISA for detection of anti-PRRSV antibodies in serum and in OF

Indirect ELISA for detection of anti-PRRSV antibodies in serum and OF were performed using Pigtype PRRS Ab and Pigtype PRRS Ab OF (Qiagen, Hilden, Germany), respectively. The result of the reaction was measured as optical density (OD) and interpreted as sample-to-positive (S/P) ratio, where S/*p* values ≥ 0.4 were considered positive, as per manufacturer’s instructions—samples deemed positive by this criterion were not retested with the same or any other method for serological detection of anti-PRRSV antibodies.

#### 2.4.4 Statistical analyses

Due to the repeated-measures structure of the data (multiple observations per individual pig within pens), generalized mixed-effects statistical models were applied to account for inherent autocorrelation (22) and to avoid pseudoreplication (23). Measurements obtained from the same individual or from pigs housed in the same pen were expected to be more similar than measurements from different individuals or pens. The random pen-effects in the model account for this effect by implying within-pen positive correlation and hence prevent type I error inflation.

Linear mixed-effects models (LME) (24) were used for continuous ELISA outcomes, while generalized linear mixed-effects models (GLMM) (25) were applied to binary PCR data as an extension of generalized linear models (GLM) (26). Because PCR outcomes followed a Bernoulli distribution, logistic regression models with random effects were employed (27). In addition, generalized additive models (GAM) (28) implemented as a GLMM framework using the *mgcv* package were used to improve computational efficiency where appropriate. All data processing and statistical analyses were performed using the R statistical computing environment (29).

## 3 Results

### 3.1 RT-PCR testing for detection of PRRSV RNA

All individual serum samples and group OF samples tested on day 0 were negative for PRRSV RNA, confirming that all gilts were PRRSV-negative upon arrival at the acclimation facilities.

#### 3.1.1 Gilt group I

In group I, PRRSV RNA was first detected in serum samples at 7 days post-exposure (DPE) in 20.0% (3/15) of gilts. By 14 DPE, the proportion of viremic animals increased to 93.3% (14/15). Viremia was not detected simultaneously in all gilts, and intermittent detection was observed in some individuals throughout the study period. At 56 DPE, 13.3% (2/15) of gilts remained viremic.

PRRSV RNA was detected in OF samples at all sampling points except 0 and 35 DPE. One gilt (individual no. 1) died before sampling at 7 DPE. A detailed timeline of individual and group results is shown in Supplementary Table S1.

#### 3.1.2 Gilt group II

In group II, PRRSV RNA was detected in serum samples of 13.3% (2/15) of gilts at 7 DPE, increasing to 86.7% (13/15) at 14 DPE. Viremia was transient in several animals, with some gilts showing viral RNA at a single sampling point only. PRRSV RNA was detected in OF samples at all time points except day 0.

During the 56-day observation period, five of 16 gilts died. The complete RT-PCR results for serum and OF samples are presented in Supplementary Table S2.

#### 3.1.3 Gilt group III

In group III, gilts were additionally exposed to ropes soaked with OF from PRRSV-positive animals after the first four sampling events (days 0, 7, 14, and 21 DPE). At 7 DPE, 62.5% (10/16) of gilts were already PRRSV RNA-positive in serum, increasing to 87.5% (14/16) at 14 DPE. Up until 28 DPE, exposure ropes were present, and group OF was concurrently collected by rope at each sampling.

Detection of PRRSV RNA in OF samples was less consistent in this group compared with the others. Viral RNA was detected in five OF samples, while the OF sample collected at 21 DPE tested negative despite concurrent viremia in 6 of 15 gilts. Three gilts died during the study; none were RT-PCR positive prior to death, and no PRRS-related cause was identified. Detailed results are shown in Supplementary Table S3.

#### 3.1.4 Gilt group IV

Similar to group III, gilts in group IV were exposed to OF-soaked ropes from PRRSV-positive pigs. As in group III, exposure ropes were present while group OF was collected at each time point. At 7 DPE, 86.7% (13/15) of gilts tested positive for PRRSV RNA in serum. At 14 and 21 DPE, 92.3% of gilts were viremic, with one gilt remaining consistently negative during this period. Four gilts died; none were RT-PCR positive before death, and diagnostic findings did not indicate PRRS-related pathology.

By the end of the study (56 DPE), only one gilt (8.3%) remained PRRSV RNA-positive. PRRSV RNA was detected in OF samples at all sampling points except 0 and 56 DPE. The complete timeline is shown in Supplementary Table S4.

#### 3.1.5 Gilt groups V and VI

Groups V and VI were housed in PRRSV-free facilities and exposed only to OF-soaked ropes originating from PRRSV-positive animals. Despite confirmed PRRSV positivity of the OF used for rope preparation, no infection occurred, PRRSV RNA was not detected in any OF samples or individual serum samples from these groups. Consequently, RT-PCR testing of individual sera was discontinued, as no evidence of infection was observed.

### 3.2 Serological detection of anti-PRRSV antibodies with indirect ELISA

All individual serum samples tested on day 0 were negative for PRRSV specific antibodies, whereas the same was not confirmed in group samples of OF – samples of OF reacted positive for presence of PRRSV specific antibodies on day 0 in gilt groups II, III and IV.

#### 3.2.1 Gilt groups I and II

In group I, PRRSV-specific antibodies were detected in one gilt (6.7%) at 7 DPE. By 14 DPE, 26.7% (4/15) of gilts had seroconverted. One gilt showed delayed seroconversion at 35 DPE. By 56 DPE, all gilts in group I were seropositive. OF samples tested positive from 14 DPE onward.

In group II, antibodies were detected in 6.7% (1/15) of gilts at 7 DPE and in 33.3% (5/15) at 14 DPE. By 21 DPE, all remaining gilts had seroconverted. OF samples were positive at all sampling points, including day 0, with S/*p* values exceeding the manufacturer’s threshold for positivity. Detailed S/p values are presented in Supplementary Tables S5, S6.

#### 3.2.2 Gilt groups III and IV

In group III, 18.8% (3/16) of gilts were seropositive at 7 DPE, increasing to 62.5% (10/16) at 14 DPE. From 21 DPE onward, all tested serum samples were positive for anti-PRRSV antibodies. OF samples were positive at all sampling points, although S/p values at 0 and 7 DPE were notably lower than at later time points.

In group IV, 92.3% (12/13) of gilts were seropositive at 14 DPE, and all remaining samples remained positive until the end of the study. OF samples were positive for antibodies throughout most of the study period, with a transient negative result observed at 7 DPE. Detailed S/p values are provided in Supplementary Tables S7, S8.

#### 3.2.3 Gilt groups V and VI

No seroconversion was observed in groups V and VI. One gilt in group VI showed a transient positive S/p value from 7 DPE onward; however, PRRSV RNA was never detected in this animal or in any other gilt from these groups. The study of these groups was therefore terminated. A prominent clinical sign of unilateral conjunctivitis was observed from the second sampling onward. Individual S/p values are available from the authors upon request.

### 3.3 Statistical analyses

Statistical analyses were performed based on treatment (TRT) groups defined by the method of PRRSV exposure. Groups I and II were combined as TRT group A (natural exposure), groups III and IV as TRT group B (natural exposure plus OF-soaked ropes), and groups V and VI as TRT group C (OF-soaked ropes only). Because acclimation in group C was unsuccessful, statistical comparisons focused primarily on TRT groups A and B (Table 3).

**Table 3:** Two-sided comparison of sera samples between groups A and B for viral RNA detection with RT-PCR.

Time (DPE)	Two-sided test comparing		*p*-value
0	A	B	1.0000
7	A	B	0.0005*
14	A	B	0.9974
21	A	B	0.1878
28	A	B	0.0318*
35	A	B	0.4491
42	A	B	0.8752
49	A	B	0.5633
56	A	B	0.9431

*Marks the statistically significant difference between the groups of gilts.

Within each TRT group, changes over time and interactions between time and treatment (time × TRT) were evaluated. The threshold for statistical significance was set at *p* < 0.05.

#### 3.3.1 Statistical analysis of RT-PCR results

RT-PCR results from OF samples were not suitable for statistical analysis due to the limited number of replicates per TRT group. Serum RT-PCR results were compared between TRT groups A and B at each sampling time point. Significant differences between groups were observed at 7 DPE (*p* = 0.0005) and 28 DPE (*p* = 0.0318), while no significant differences were detected at other time points (Supplementary Table S11).

#### 3.3.2 Statistical analysis of ELISA results

ELISA results were analyzed separately for serum and OF samples and in comparison with each other. Linear mixed-effects modeling revealed a highly significant effect of time on antibody detection in both serum and OF samples (*p* < 0.001). The interaction between time and treatment (time × TRT) was also significant for both matrices, whereas the effect of treatment alone was not significant (Table 4).

**Table 4:** The significance of each effect on specific antibody detection in sera and in OF.

Sample type	Effect	*p*-value
Serum	Time	<0.001*
	TRT	0.827
	Time × TRT	<0.001*
OF	Time	<0.001*
	TRT	0.926
	Time × TRT	0.047*

*Marks the statistically significant of the effect on specific antibody detection.

Pairwise comparisons of serum samples between TRT groups A and B demonstrated significant differences in antibody detection at 14 DPE (*p* < 0.0001), 21 DPE (*p* = 0.0001), and 49 DPE (*p* = 0.0025) (Table 5).

**Table 5:** Two-sided comparison of sera samples between gilt groups A and B for specific antibody detection with ELISA.

Time (DPE)	Two-sided test comparing		*p*-value
0	A	B	0.6198
7	A	B	0.6721
			
14	A	B	<0.0001*
21	A	B	0.0001*
28	A	B	0.0748
35	A	B	0.9780
42	A	B	0.7127
49	A	B	0.0025*
56	A	B	0.0857

*Marks the statistically significant difference between the groups of gilts.

## 4 Discussion

Given the substantial impact of PRRS on animal health and production economics, effective disease management strategies are essential. In production systems where strict biosecurity measures—such as complete separation of pig categories and all-in/all-out management—cannot be fully implemented, PRRS control rather than elimination often represents the most realistic option (30). Replacement gilts play a pivotal role in PRRS control programs and must be adequately acclimated before introduction into the breeding herd and first gestation (15).

The commercial farm included in this study relies on natural exposure for gilt acclimation. Although widely practiced, this approach lacks control over the timing and dose of infection and the host response. To address these limitations, we evaluated the use of PRRSV-contaminated material—cotton ropes soaked in OF from PRRSV-infected pigs—as an additional or alternative infectious stimulus. We further assessed whether acclimation could be achieved using OF-soaked ropes alone and monitored the acclimation process using both individual serum samples and group OF samples.

Natural exposure is associated with uncertainty regarding infection dynamics, and acclimation using this method typically requires at least 3 months (31). We hypothesized that the addition of a more standardized exposure method could reduce this uncertainty by inducing earlier infection, shortening convalescence, and promoting the development of homologous immunity. To our knowledge, this study is the first to systematically evaluate the use of PRRSV-contaminated ropes for gilt acclimation, as no peer-reviewed data on this approach has previously been published.

Neutralizing antibodies against PRRSV generally appear no earlier than 28 DPE and contribute to viral clearance from the bloodstream (32). Accordingly, comparisons among treatment groups—natural exposure only (groups I and II; treatment group A), natural exposure plus OF-soaked ropes (groups III and IV; treatment group B), and OF-soaked ropes only (groups V and VI; treatment group C)—were central to evaluating the effectiveness of the different acclimation strategies. However, these observations are based on isolated time-point differences and were not consistently maintained across the entire study period, and we therefore interpret them with caution to avoid over-extrapolating their biological significance.

The absence of PRRSV RNA detection and seroconversion in treatment group C indicates that OF-soaked ropes alone did not provide sufficient infectious stimulus to establish infection. The single gilt in group VI with a positive ELISA result despite consistently negative RT-PCR findings may reflect either a false-positive serological reaction or limited immune stimulation without productive infection. The same applies for gilt number 3 from group III, who tested seropositive result on 0 DPE (S/*p* value of 0.40 in serum sample), but was RT-PCR was negative upon arrival. Because no confirmatory testing or serial dilution was conducted, this isolated positive S/p value was interpreted cautiously and considered biologically implausible in the absence of viral RNA detection in any matrix. Occasional transient ELISA reactivity has been reported previously in PRRSV-negative animals and may arise from non-specific binding, assay variability near the cut-off, or individual immune characteristics. Individual variability in immune responses—driven by genetic background, rearing conditions, and immune competence, including local mucosal immunity in the oral cavity—may contribute to such findings (33–35).

Although PRRSV is known to be transmitted efficiently via various excreta (8), our results suggest that OF alone does not consistently contain a sufficient amount of infectious virus to induce infection. In contrast, several statistically significant differences between treatment groups A and B indicate that the additional viral stimulus provided by OF-soaked ropes influenced infection onset and initial detection. Earlier detection of viremia at 7 DPE (*p* < 0.0005) and earlier absence of detectable viremia at 28 DPE (*p* = 0.0318) were observed in treatment group B. However, complete elimination of viremia was not achieved in all gilts in any group, and no differences between treatments were observed at the end of the study. This finding contrasts with reports by Batista et al. (36), who described complete clearance of viremia by 30 DPE, underscoring variability in PRRSV infection dynamics across studies and production systems.

Antibody production followed the expected temporal pattern, with initial seroconversion detected as early as 7 DPE and most animals seropositive by 14 DPE (4, 37). Treatment group B showed a significantly higher proportion of seroconverted gilts than treatment group A at early time points (*p* < 0.0001), indicating that the additional infectious stimulus enhanced humoral responses. Differences in S/*p* values between groups at 21 DPE (*p* < 0.0001) and 49 DPE (*p* = 0.0025) further support this conclusion. However, these differences did not translate into reduced viremia at the end of the study, highlighting that PRRSV elimination from the bloodstream is not solely dependent on antibody-mediated immunity but also involves other immune mechanisms that were not evaluated here (32).

Statistical analyses showed no significant differences between serum and OF samples in their ability to detect PRRSV, supporting the utility of OF as a diagnostic matrix (20, 38). Nevertheless, several discrepancies between serum and OF RT-PCR results were observed, including sampling points where OF tested negative despite concurrent viremia in multiple gilts (notably in group III). These findings reflect known differences in viral load dynamics and detection sensitivity between matrices. OF viral RNA levels are influenced by chewing behavior, dilution effects, and environmental degradation, making OF particularly susceptible to false-negative results when viral loads approach the assay’s detection limit. Intermittent positive–negative–positive RT-PCR patterns observed in individual serum samples likely reflect variability near the detection threshold of endpoint RT-PCR rather than true biological fluctuations, and this methodological limitation is now acknowledged in the manuscript. This finding suggests differences in viral kinetics between serum and OF, even though PRRSV has been reported to persist longer in OF in some cases (39).

Detection of PRRSV-specific antibodies in OF remains challenging, despite the availability of standardized commercial assays (40–42). Unexpected positive OF results at 0 DPE prompted consultation with the assay manufacturer, who acknowledged known limitations and indicated withdrawal of the product from the market. Retesting selected samples with an alternative commercial assay (IDEXX PRRSV Oral Fluid Antibody Test) yielded comparable results. These low-level OF signals at day 0 occurred despite all sera testing negative and were interpreted as non-specific reactions rather than evidence of prior exposure. While OF is generally considered resistant to external contamination, pre-analytical factors such as collection, transport, and storage may influence test outcomes (43, 44). Although correction of S/p values has been proposed to address underestimation of antibody levels in OF (45–47), this approach was not directly applicable to the present dataset and warrants further investigation.

This study was conducted under field conditions, and although gilts originated from a PRRSV-negative source herd and were vaccinated against PCV2 prior to transfer, systematic testing for other respiratory pathogens during the acclimatization period was not performed. Therefore, the potential presence of concurrent infections represents a limitation of the study and may have influenced the clinical expression and viral dynamics of PRRSV, which should be considered when interpreting the results.

In conclusion, the addition of PRRSV-contaminated OF-soaked ropes to natural exposure accelerated early infection and enhanced antibody responses during gilt acclimation but was insufficient as a standalone method to induce infection. While the use of OF-soaked ropes represents a promising supplementary tool for improving the predictability of PRRS acclimation, complete control of viremia remains influenced by complex host–pathogen interactions beyond humoral immunity alone. Oral fluid proved to be a useful diagnostic sample for monitoring PRRSV infection; however, differences in sensitivity between matrices, particularly near the detection limit, and challenges related to antibody detection highlight important diagnostic limitations that should be considered when interpreting OF-based surveillance results. These findings contribute to a better understanding of gilt acclimation strategies under field conditions and provide a basis for refining practical PRRSV control measures in the context of ongoing viral circulation in the face of ongoing viral evolution. Improving gilt acclimation strategies represents a critical component of PRRSV control programs and may contribute to long-term efforts aimed at reducing viral circulation and facilitating herd-level elimination.

## Data Availability

The raw data supporting the conclusions of this article will be made available by the authors, without undue reservation.
