# Supplementary material for: The applicability of pig oral fluid in laboratory diagnostics of porcine reproductive and respiratory syndrome and its effectiveness in controlled exposure of gilts
Source: Front Vet Sci. 2026 Mar 6;13:1795728. doi: 10.3389/fvets.2026.1795728 (PMC13002400; doi:10.3389/fvets.2026.1795728)
Supplement: Supplementary file 1 [file Data_Sheet_1.PDF]

Supplementary Table 1: Results of sera and OF testing for PRRSV RNA with RT-PCR in group I. Positive results are colored red.

	DPE								
Individual gilt number(serum)	0	7	14	21	28	35	42	49	56
1	neg								
2	neg	neg	pos	pos	pos	pos	neg	neg	neg
3	neg	neg	neg	pos	pos	neg	pos	neg	neg
4	neg	neg	pos	pos	neg	pos	pos	neg	neg
5	neg	pos	pos	pos	pos	neg	neg	neg	neg
6	neg	neg	pos	pos	pos	neg	neg	neg	neg
7	neg	neg	pos	pos	pos	neg	neg	neg	neg
8	neg	neg	pos	neg	pos	pos	pos	neg	pos
9	neg	neg	pos	pos	pos	pos	neg	neg	neg
10	neg	pos	pos	pos	pos	pos	neg	neg	neg
11	neg	neg	pos	pos	pos	pos	neg	neg	neg
12	neg	neg	pos	pos	pos	pos	neg	neg	neg
13	neg	neg	pos	pos	neg	neg	neg	pos	neg
14	neg	neg	pos	pos	pos	pos	neg	neg	neg
15	neg	neg	pos	neg	neg	neg	neg	pos	neg
16	neg	pos	pos	pos	pos	pos	neg	neg	pos
pos/all	0/16	3/15	14/15	13/15	12/15	9/15	3/15	2/15	2/15
% pos	0	20,0	93,0	86,0	80,0	60,0	20,0	13,3	13,3
OF	neg	pos	pos	pos	pos	neg	pos	pos	pos

Supplementary Table 2: Results of sera and OF testing for PRRSV RNA with RT-PCR in group II. Positive results are colored red.

	DPE								
Individual gilt number(serum)	0	7	14	21	28	35	42	49	56
1	neg	neg	pos	pos					
2	neg	neg	pos	neg	neg	neg	neg	neg	pos
3	neg	neg	pos	pos	pos				
4	neg	neg	pos	pos	pos	pos	pos	pos	
5	neg	neg	pos	pos	pos	neg	pos	neg	neg
6	neg	pos	pos	neg	pos	neg	neg	neg	neg
7	neg	neg	pos	pos	pos	neg	neg	pos	neg
8	neg								
9	neg	neg	neg	pos	pos	neg	neg	neg	neg
10	neg	neg	pos	pos	pos	neg	neg	neg	neg
11	neg	neg	neg	neg	neg	pos	neg	neg	neg
12	neg	neg	pos	pos	neg	pos	neg	neg	pos
13	neg	neg	pos	pos	pos	neg	neg	pos	neg
14	neg	neg	pos						
15	neg	pos	pos	pos	neg	neg	pos	neg	neg
16	neg	neg	pos	pos	neg	neg	neg	neg	neg
pos/all	0/16	2/15	13/15	11/14	9/13	3/12	3/12	3/12	2/11
% pos	0	13,3	86,7	78,6	69,2	25,0	25,0	25,0	18,2

OF	neg	pos	pos	pos	pos	pos	pos	pos	pos

Supplementary Table 3: Results of sera and OF testing for PRRSV RNA with RT-PCR in group III. Positive results are colored red.

	DPE								
Individual gilt number(serum)	0	7	14	21	28	35	42	49	56
1	neg	pos	pos	neg	pos	neg	neg	neg	neg
2	neg	neg	pos	neg	neg	pos	neg	neg	pos
3	neg	pos	neg	neg	neg	neg	neg	neg	neg
4	neg	pos	pos	neg	neg	neg	neg	neg	neg
5	neg	pos	pos	pos	neg	neg	neg	neg	neg
6	neg	neg	pos						
7	neg	pos	neg	neg					
8	neg	neg	pos	neg	pos	pos	neg	neg	neg
9	neg	pos	pos	neg	neg	pos	neg	neg	neg
10	neg	pos	pos	pos	neg	pos	neg	neg	neg
11	neg	pos	pos	neg	pos	neg	neg	neg	neg
12	neg	neg	pos	pos	neg	neg	neg	neg	neg
13	neg	pos	pos	pos	pos	neg	neg	neg	pos
14	neg	neg	pos	pos					
15	neg	neg	pos	pos	pos	neg	pos	pos	pos
16	neg	neg	pos	neg	pos	neg	neg	neg	neg
pos/all	0/16	10/16	14/16	6/15	6/13	4/13	1/13	1/13	3/13
% pos	0	62,5	87,5	40,0	46,2	30,8	7,7	7,7	23,1
OF	neg*	pos*	pos*	neg*	pos	neg	neg	pos	neg

*gilts were exposed to ropes soaked in OF of PRRS positive animals after the sampling

Supplementary Table 4: Results of sera and OF testing for PRRSV RNA with RT-PCR in group IV. Positive results are colored red.

	DPE								
Individual gilt number(serum)	0	7	14	21	28	35	42	49	56
1	neg	neg	pos	pos	neg	neg	pos	pos	pos
2	neg	pos	pos	pos	pos	pos	neg	neg	neg
3	neg	neg	pos	pos	neg	pos	neg	neg	neg
4	neg								
5	neg	pos	pos	pos	neg	neg	pos	neg	neg
6	neg	pos	pos	pos	neg	neg	neg	neg	neg
7	neg	pos							
8	neg	pos	pos	pos	pos	neg	neg	pos	neg
9	neg	pos	pos	pos	neg	neg	neg	neg	neg
10	neg	pos	neg	neg	pos	neg	neg	neg	neg
11	neg	pos	pos	pos	neg	neg	neg	neg	neg
12	neg	pos	pos	pos	neg	neg	pos	neg	neg
13	neg	pos	pos	pos					
14	neg	pos							

15	neg	pos	pos	pos	neg	pos	neg	neg	neg
16	neg	pos	pos	pos	neg	pos	pos	neg	neg
pos/all	0/16	13/15	12/13	12/13	3/12	4/12	4/12	2/12	1/12
% pos	0	86,7	92,3	92,3	25,0	33,3	33,3	16,7	8,3
OF	neg	pos	pos	pos	pos	pos	pos	pos	neg

*gilts were exposed to ropes soaked in OF of PRRS positive animals after the sampling

Supplementary Table 5: S/P ratio of individual serum samples, their mean value and S/P values of group OF samples of group I. Positive results are colored red.

		DPE								
		0	7	14	21	28	35	42	49	56
Individual gilt number(serum)	1	0,15								
	2	0,08	0,26	0,06	2,24	2,87	2,43	1,07	2,47	3,45
	3	-0,13	0,01	-0,03	2,45	2,69	2,64	2,00	2,14	2,53
	4	0,15	0,19	0,35	1,79	2,55	2,12	0,61	2,03	2,14
	5	0,35	0,43	1,93	2,78	2,17	1,76	1,30	1,85	1,78
	6	0,14	0,03	-0,01	2,34	2,46	2,19	1,85	1,29	1,86
	7	-0,14	0,04	2,38	3,21	2,52	2,01	1,88	2,34	1,73
	8	-0,19	0,04	1,30	2,39	2,76	2,25	2,49	1,80	2,74
	9	0,34	0,20	0,17	2,82	3,22	3,02	2,47	2,61	3,06
	10	0,17	0,21	0,12	2,91	3,00	2,21	1,70	1,58	2,30
	11	-0,08	-0,01	0,03	2,57	3,03	2,68	1,73	2,22	2,92
	12	0,05	0,20	0,07	2,30	2,87	2,53	1,73	1,82	3,27
	13	0,22	0,22	0,25	2,74	2,75	2,68	2,43	2,42	3,24
	14	0,10	0,03	0,03	0,05	0,00	2,13	1,62	1,49	2,21
	15	0,02	0,05	0,01	2,98	3,01	2,73	2,45	1,48	3,10
	16	0,12	0,22	0,56	2,53	2,68	2,40	1,66	2,56	1,69
	pos/all	0/16	1/15	4/15	14/15	14/15	15/15	15/15	15/15	15/15
	% pos	0	6,7	26,7	93,3	93,3	100	100	100	100
	$\overline{S/P}$	0,08	0,14	0,48	2,41	2,57	2,39	1,80	2,01	2,53
	OF	0,29	0,20	2,42	4,33	5,38	6,17	4,21	6,49	3,14

Supplementary Table 6: S/P ratio of individual serum samples, their mean value and S/P values of group OF samples of group II. Positive results are colored red.

		DPE								
		0	7	14	21	28	35	42	49	56
Individual gilt number(serum)	1	0,35	0,46	0,01	2,60					
	2	-0,01	0,04	0,01	2,45	2,28	2,28	2,46	2,29	1,77
	3	0,08	0,15	0,31	0,66	2,16				
	4	0,18	0,14	0,20	2,42	2,56	2,53	2,70	2,61	
	5	0,11	0,37	1,07	1,72	2,45	2,23	2,14	3,32	2,55
	6	0,29	0,42	1,08	2,44	2,44	2,71	2,42	3,52	2,45
	7	0,11	0,23	0,45	1,78	2,42	2,82	2,13	3,00	1,98
	8	-0,14								
	9	0,26	0,11	0,31	1,58	2,91	2,76	2,00	3,06	1,85

10	0,12	0,03	0,01	2,50	1,34	2,70	2,94	3,23	2,13
11	0,15	-0,02	0,10	2,58	2,49	2,43	1,88	2,56	1,85
12	0,05	0,07	0,40	2,44	2,28	2,36	2,69	2,52	1,93
13	0,20	0,12	0,22	2,20	2,53	2,38	1,97	3,06	1,80
14	0,24	0,16	0,28						
15	0,17	0,21	2,49	3,05	2,56	2,55	2,52	2,65	1,84
16	0,10	0,21	0,45	1,85	2,41	2,36	2,58	1,93	1,27
pos/all	0/16	1/15	5/15	14/14	13/13	12/12	12/12	12/12	11/11
% pos	0	6,7	33,3	100	100	100	100	100	100
$\overline{S/P}$	0,14	0,18	0,49	2,16	2,37	2,51	2,37	2,81	1,95
OF	0,40	1,18	3,26	6,14	5,23	4,86	3,18	3,05	3,04

Supplementary Table 7: S/P ratio of individual serum samples, their mean value and S/P values of group OF samples of group III. Positive results are colored red.

	DPE								
Individual gilt number(serum)	0	7	14	21	28	35	42	49	56
1	0,10	0,38	1,41	3,11	2,46	2,91	2,53	2,43	2,62
2	0,19	-0,02	0,07	3,12	2,72	2,95	2,05	1,71	1,38
3	0,40	0,52	2,43	2,87	2,48	2,94	1,97	1,70	1,46
4	0,09	0,09	2,70	3,58	2,85	3,12	2,16	2,20	1,66
5	0,24	0,09	1,82	2,97	2,17	2,56	2,04	2,22	2,41
6	0,12	-0,02	0,34						
7	0,00	0,08	0,27	3,09					
8	-0,01	0,44	2,18	3,22	2,45	3,13	2,06	1,95	1,63
9	0,14	0,01	1,82	3,16	1,89	3,07	1,88	1,99	2,08
10	0,06	0,12	0,46	2,93	2,33	2,26	1,52	1,72	2,06
11	0,12	-0,06	2,25	2,74	2,16	2,17	1,87	1,74	2,31
12	0,01	-0,01	0,37	2,99	2,40	2,14	1,55	1,42	1,88
13	0,01	0,05	0,16	3,66	2,55	2,44	1,65	1,59	1,48
14	0,08	0,49	2,61	2,95					
15	0,03	0,00	0,18	2,90	2,64	2,64	2,05	1,94	2,24
16	0,07	-0,04	1,54	3,20	2,23	2,51	1,81	1,90	1,15
pos/all	0/16	3/16	10/16	15/15	13/13	13/13	13/13	13/13	13/13
% pos	0	18,8	62,5	100	100	100	100	100	100
$\overline{S/P}$	0,10	0,13	1,29	3,10	2,41	2,68	1,93	1,89	1,87
OF	0,51*	0,53*	5,89*	6,76*	4,55	4,84	2,97	3,61	4,12

*gilts were exposed to ropes soaked in OF of PRRS positive animals after the sampling

Table 8: S/P ratio of individual serum samples, their mean value and S/P values of group OF samples of group IV. Positive results are colored red.

	DPE								
Indivi dual gilt numbe [illegible]	0	7	14	21	28	35	42	49	56
1	0,00	0,03	0,30	1,95	2,27	1,56	1,50	1,61	1,69
2	0,12	0,19	2,68	2,67	2,81	1,76	2,00	2,24	2,39
3	-0,05	-0,03	0,44	1,87	2,96	1,98	1,80	1,60	2,18

4	-0,05								
5	0,03	0,07	2,28	2,64	3,41	2,55	2,54	2,71	2,83
6	-0,04	-0,01	2,01	1,74	3,07	2,02	1,97	1,86	2,02
7	0,20	0,20							
8	0,04	0,07	0,82	1,94	2,49	2,10	1,64	1,61	1,95
9	0,03	0,15	2,95	2,74	3,32	2,51	2,47	2,67	1,94
10	-0,08	-0,01	1,59	2,27	2,63	2,03	1,65	1,36	1,49
11	-0,07	-0,09	2,92	2,35	3,34	2,33	2,29	2,46	2,61
12	-0,02	0,12	2,01	2,44	3,24	2,30	2,14	2,29	2,42
13	-0,01	0,06	1,04	2,00					
14	0,04	0,47							
15	0,10	0,12	3,19	2,40	3,38	2,46	2,35	2,49	2,97
16	0,00	0,05	0,99	2,95	3,15	2,61	2,68	2,62	3,13
pos/all	0/16	1/15	12/13	13/13	12/12	12/12	12/12	12/12	12/12
% pos	0	6,7	92,3	100	100	100	100	100	100
$\overline{S/P}$	0,02	0,09	1,79	2,30	3,01	2,19	2,09	2,13	2,30
OF	0,82*	0,13*	0,65*	3,47*	3,82	3,43	4,00	2,23	2,54
